# Inflammatory Cytokines Associate With Neuroimaging After Acute Mild Traumatic Brain Injury

**DOI:** 10.3389/fneur.2020.00348

**Published:** 2020-05-19

**Authors:** Katie A. Edwards, Cassandra L. Pattinson, Vivian A. Guedes, Jordan Peyer, Candace Moore, Tara Davis, Christina Devoto, L. Christine Turtzo, Lawrence Latour, Jessica M. Gill

**Affiliations:** ^1^National Institutes of Health, National Institute of Nursing Research, Bethesda, MD, United States; ^2^The Henry M. Jackson Foundation for the Advancement of Military Medicine, Bethesda, MD, United States; ^3^Center for Neuroscience and Regenerative Medicine, Bethesda, MD, United States; ^4^Johns Hopkins Suburban Hospital, Bethesda, MD, United States; ^5^National Institutes of Health, National Institute of Neurological Disorders and Stroke, Bethesda, MD, United States; ^6^Center for Neuroscience and Regenerative Medicine, Uniformed Services University of the Health Sciences, Biomarker Core, Bethesda, MD, United States

**Keywords:** cytokines, neuroimaging, mild traumatic brain injury, inflammation, cardiovascular disease risk

## Abstract

**Introduction:** Elevated levels of blood-based proinflammatory cytokines are linked to acute moderate to severe traumatic brain injuries (TBIs), yet less is known in acute mild (m)TBI cohorts. The current study examined whether blood-based cytokines can differentiate patients with mTBI, with and without neuroimaging findings (CT and MRI).

**Material and Methods:** Within 24 h of a mTBI, determined by a Glasgow Coma Scale (GCS) between 13 and 15, participants (*n* = 250) underwent a computed tomography (CT) and magnetic resonance imaging (MRI) scan and provided a blood sample. Participants were classified into three groups according to imaging findings; (1) CT+, (2) MRI+ (CT–), (3) Controls (CT– MRI–). Plasma levels of circulating cytokines (IL-6, IL-10, TNFα), and vascular endothelial growth factor (VEGF) were measured using an ultra-sensitive immunoassay.

**Results:** Concentrations of inflammatory cytokines (IL-6, TNFα) and VEGF were elevated in CT+, as well as MRI+ groups (*p* < 0.001), compared to controls, even after controlling for age, sex and cardiovascular disease (CVD)-related risk factors; hypertension, and hyperlipidemia. Post-concussive symptoms were associated with imaging groupings, but not inflammatory cytokines in this cohort. Levels of VEGF, IL-6, and TNFα differentiated patients with CT+ findings from controls, with the combined biomarker model (VEGF, IL-6, TNFα, and IL-10) showing good discriminatory power (AUC 0.92, 95% CI 0.87–0.97). IL-6 was a fair predictor of MRI+ findings compared to controls (AUC 0.70, 95% CI 0.60–0.78). Finally, the combined biomarker model discriminated patients with MRI+ from CT+ with an AUC of 0.71 (95% CI 0.62–0.80).

**Conclusions:** When combined, IL-6, TNFα, and VEGF may provide a promising biomarker cytokine panel to differentiate mTBI patients with CT+ imaging vs. controls. Singularly, IL-6 was a fair discriminator between each of the imaging groups. Future research directions may help elucidate mechanisms related to injury severity and potentially, recovery following an mTBI.

## Introduction

Mild traumatic brain injury (mTBI) is common, affecting over 42 million people worldwide each year; accounting for 80–90% of all head injury cases (1–3). Short and long-term neurological, cognitive and psychiatric symptoms have been associated with mTBI, representing a significant burden to patients, families, and the public health system (3). After sustaining a mTBI, patients commonly display non-specific post-concussive symptoms, including headaches, vision and balance impairments, poor attention, and irritability (4, 5). Although most of the individuals with mTBI fully recover, a subset of patients develop persistent symptoms (6, 7). Identifying differences in the pathophysiology of those who may develop persistent symptoms may help identify novel therapeutic avenues. Evidence implicates that individuals with complicated mTBI (presence of intracranial abnormality on CT) may experience increased or persistent symptoms as compared to individuals without intracranial abnormalities on imaging (8–11). With the recently FDA-approved biomarkers glial fibrillary acidic protein (GFAP) and ubiquitin C-terminal hydrolase (UCH-L1) to aid in clinical CT decisions (12), efforts to identify these patients at risk are underway. However, TBIs initiate a number of secondary pathological processes including inflammation (13), and examination of related blood-based biomarkers may help specify pathological pathways for future research and therapeutic applications.

TBIs trigger inflammatory activity, initiating a pronounced increase in cytokines within 24 h after the head injury among patients with all TBI severities that are important for recovery processes (14–16). Pro- and anti-inflammatory cytokines are essential to coordinating a balanced inflammatory response following TBI (13). Interleukin (IL)-6 activity is crucial for immune cell recruitment in the acute phase of TBI, although IL-6 deficiency as well as overexpression are detrimental in preclinical models (17–19). Deficiency in interleukin (IL)-10, which functions in a neuroprotective role, also results in poor outcomes in preclinical models (20). Tumor necrosis factor alpha (TNFα) mediates the inflammatory response through microglial activation and increased chemokine production, and preclinical models demonstrate neuronal damage with elevated TNFα after TBI (21–23). For individuals with moderate or severe TBIs, worse clinical outcomes have been linked to increased acute levels of IL-6, IL-10, and TNFα (24–28). Elevations in peripheral levels of IL-6 and TNFα have been reported following blast exposure in military personnel (29), suggesting that mild brain injuries may have similar biomarker changes. One preliminary study of extracellular vesicles observed elevated TNFα following sports-related concussions (30). Elevated plasma levels of the IL-6 and TNFα are reported in military personnel with mild blunt force and/or blast TBIs, and these elevations remain in personnel with neurological symptoms (31). IL-6 is observed to be acutely increased following mTBI in emergency room patients (32) and also significantly discriminated athletes with concussion from controls within 6 h of injury and was associated with post-concussive symptoms after injury (33).

A critical issue is that typically studies of inflammatory cytokines following TBIs do not account for individuals who have pre-existing inflammatory cardiovascular disease (CVD) risk factors, including smoking, hyperlipidemia, and hypertension. Elevations in blood levels of several proinflammatory cytokines, including IL-6 and TNFα, relate to risk of coronary heart disease in clinical populations (34). In preclinical mTBI models, cerebrovascular dysfunction is linked to compromised immune functioning, as well as neurobehavioral deficits (35). Thus, determining the impact of CVD risk factors is important, as they may alter any observed inflammatory responses following mTBI and/or present additional patient burden which may impede recovery. One protein which has been shown to modulate inflammatory responses following TBI and has been linked to regulation of permeability in vasculature is vascular endothelial growth factor (VEGF) (36–40). In a study of patients with TBI, ranging from mild to severe, Li et al. (38) found that lower VEGF levels at day 7, as well as higher levels at 21 days post-injury were associated with improved health. However, examining the effects of VEGF acutely post-injury and how this response may be associated with injury severity remains to be determined.

To address these critical issues, we analyzed the relationship between peripheral blood levels of the cytokines IL-6, IL-10, TNFα, and VEGF and neuroimaging findings acutely following a mTBI. We evaluated associations between demographic and clinical data, and neuroimaging results, examining CVD history and risk factors when considering correlations between inflammatory cytokines and mTBI.

## Materials and Methods

### Participants

Participants were enrolled into the Traumatic Head Injury Neuroimaging Classification (THINC) protocol NCT01132937 and protocol 09-NR-0131 at emergency departments in the Washington DC metropolitan area. Both protocols were approved by the National Institutes of Health Intramural Institutional Review Board (IRB). Prior to participation in the study, written informed consent was obtained from all participants. Informed consent for non-English speaking participants was obtained using an IRB-approved translated consent document in a language that the participant understood as well as oral translation by a qualified translator. If there was any indication that the participant did not understand, the participant was not enrolled in the study. Study inclusion criteria included: (1) mild TBI, (2) 18–96 years of age, (3) initial Glasgow Coma Scale of > 13 in the emergency department, (4) collection of blood specimens, and (5) initial imaging (clinical CT and research MRI) completed within 24 h of injury. Exclusion criteria included unstable psychiatric conditions, contraindications to MRI scanning or conditions which would preclude entry to MRI scanner, and pregnancy. Post-concussive symptoms were assessed using the Neurobehavioral Symptom Inventory (NSI) during the emergency department visit, within 24 h of injury. The NSI is a 22-item assessment of somatosensory, affective, cognitive, and vestibular symptoms, with ratings from 0 (none) to 4 (very severe) for each item (41, 42). Participants also self-reported any current or past history with each of the three major risk factors of CVD disease; smoking, hypertension, and hyperlipidemia (43).

### Imaging Protocol

MRI was completed using a 3T (Siemens Healthcare, Malvern, PA). MRI imaging protocol included diffusion-tensor imaging, T2^*^ gradient recall echo imaging, susceptibility weighted imaging, 3D high-resolution T1 (3DT1), dynamic susceptibility contrast perfusion-weighted imaging, and pre- and post-contrast T1 and T2-Fluid-attenuated inversion recovery (FLAIR). Clinical CT was performed within 24 h of injury, using a standard protocol. Participants with mTBI were categorized into three groups depending on the results of neuroimaging: (1) CT positive, complicated mTBI (CT+; *n* = 64); (2) MRI positive [MRI+ (CT–); *n* = 80]; and (3) control group (CT– and MRI–; *n* = 106).

### Laboratory Methods

Blood specimens were collected into ethylenediaminetetraacetic acid tubes, centrifuged, aliquoted for plasma, and stored at −80°C. Blinded to the subject's clinical history and imaging data, plasma samples were analyzed using Simoa (Single Molecule Array) Neurology 4-plex assay kit (Quanterix, Lexington, MA) for the measurement of IL-6, IL-10, TNFα, and VEGF.

### Statistical Analyses

Analyses were conducted using SPSS V24.0 (Armonk, NY: IBM Corp.) and GraphPad Prism 7.04 (La Jolla, CA: GraphPad Software). Analysis of variance (ANOVA) and Chi-square tests were performed to determine group differences on demographic characteristics and CVD risk factors. Analysis of covariance (ANCOVA) were subsequently run, controlling for significant factors; hyperlipidemia, hypertension, sex, and age. Binomial logistic regression provided the individual VEGF, IL-6, IL-10, TNFα, and combined biomarker model ROC curves. Due to significant age variances across the groups, the ROC curves were adjusted for age.

## Results

Demographic and clinical characteristics of study participants are described in Table 1. Participants were mostly male (72.4%), aged between 18 and 96 years (*M* = 46.1 years; *SD* = 17.9). There were no significant differences observed in race or ethnicity between the groups. Participants with CT+ imaging findings were significantly older than controls (*p* < 0.001); MRI+ did not differ significantly between any of the groups. CT+ patients were significantly more likely to have hypertension and hyperlipidemia then both the MRI+ and controls. Age, sex, hyperlipidemia, and hypertension were controlled for in subsequent statistical analyses (Table 1).

**Table 1:** Demographic and clinical data.

	**CT+ (*n* = 64)**	**MRI+(*n* = 80)**	**Control (CT– and MRI–, *n* = 106)**	***p***
**Age in years, M** ***(SD)***	54.0 (22.4)	44.7 (16.7)	42.3 (15.7)	0.001
**Sex, No. (%)**				0.008
Male	52 (81.3)	63 (78.8)	66 (62.3)	
Female	12 (18.8)	17 (21.3)	40 (37.7)	
**Race, No. (%)**				0.948
White/Caucasian	44 (68.8)	60 (75.0)	71 (67.0)	
Black/African American	17 (26.6)	17 (21.2)	28 (26.4)	
Asian	2 (3.1)	2 (2.5)	3 (2.8)	
Multiple races	1 (1.6)	1 (1.3)	1 (0.9)	
Other	0 (0)	0 (0)	3 (2.8)	
**Ethnicity, No. (%)**				
Latino/Hispanic	9 (14.1)	13 (16.3)	26 (24.5)	0.289
**NSI Symptoms, M** ***(SD)***	16.6 (13.5)	15.5 (10.9)	20.4 (16.4)	0.047
**CVD Risk Factors, yes %**				
Smoking	34.4	25.0	24.5	0.326
Hyperlipidemia	43.8	21.5	15.5	<0.001
Hypertension	39.7	24.4	35.9	0.003

*CT, computed tomography; CVD, cardiovascular disease; M, mean; MRI, magnetic resonance imaging; NSI, Neurobehavioral Symptom Inventory; SD, standard deviation*.

**Figure 1:**
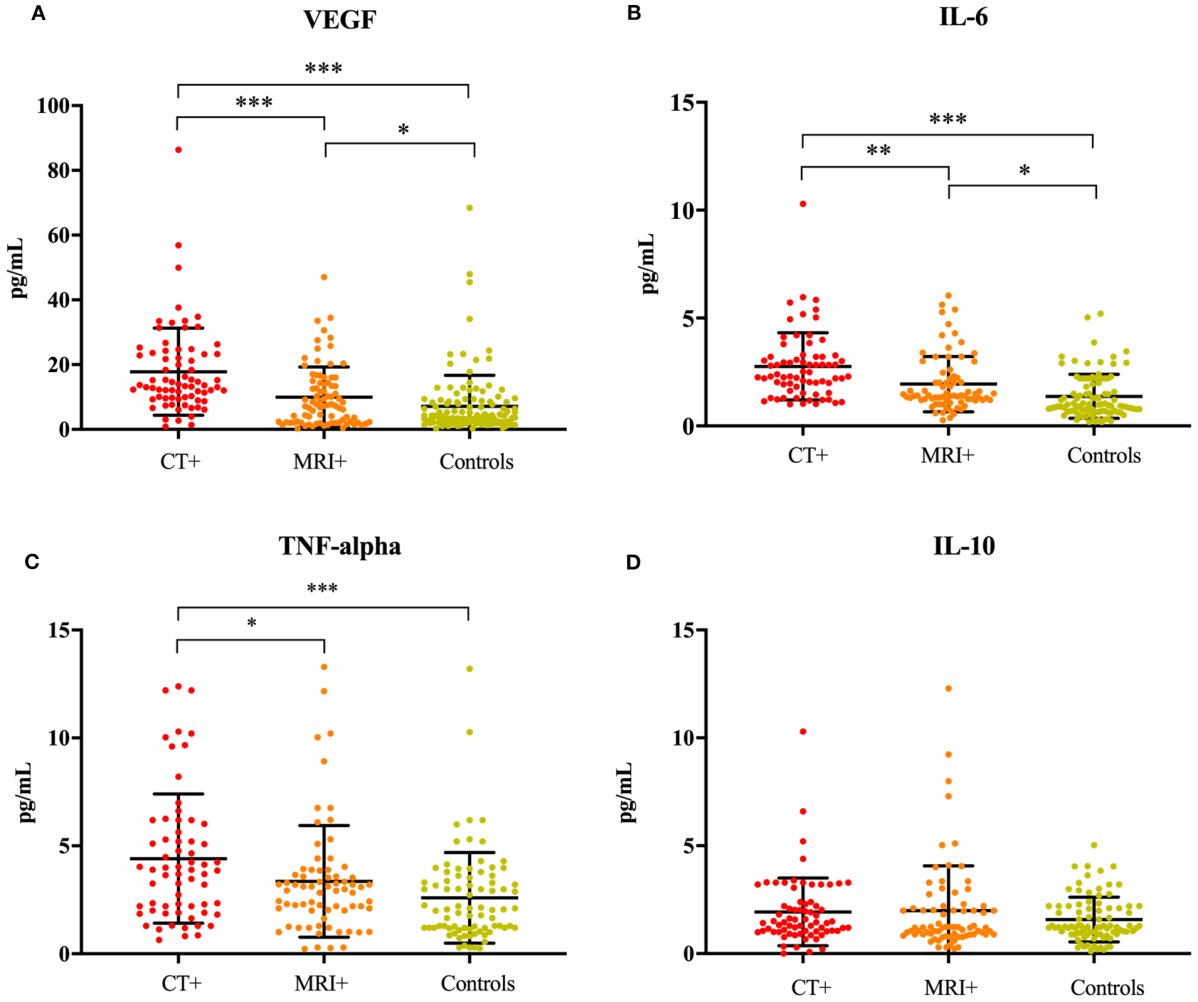
Inflammatory cytokines are associated with neuroimaging. Dot plots showing **(A)** VEGF, **(B)** IL-6, **(C)** TNFα, and **(D)** IL-10 concentrations in the CT+, MRI+, and control groups. Significant differences are indicated with *p < 0.05, **p < 0.01, and ***p < 0.001. VEGF, vascular endothelial growth factor; IL-6, interleukin 6; TNFα, tumor necrosis factor alpha; IL-10, interleukin 10.

The ANCOVA models were significant for IL-6 [*F*_(6, 205)_ = 11.56, *p* < 0.001], TNFα [*F*_(6, 181)_ = 4.29, *p* < 0.001], VEGF [*F*_(6, 234)_ = 11.84, *p* < 0.001], and the IL-6/IL-10 ratio [*F*_(6, 199)_ = 4.71, *p* < 0.001]. The model for IL-10 was not significant (*p* = 0.828). Concentrations of IL-6 [*F*_(2, 205)_ = 29.21, *p* < 0.001], TNFα [*F*_(2, 181)_ = 9.28, *p* < 0.001], VEGF [*F*_(2, 234)_ = 31.98, *p* < 0.001], and the IL-6/IL-10 ratio [*F*_(2, 199)_ = 9.38, *p* < 0.001] significantly differed across imaging groups, even after controlling for age, sex, hyperlipidemia and hypertension (Figure 1). IL-6 was significantly higher in the CT+ group compared to the MRI+ (*p* = 0.001) and control (*p* < 0.001) groups (Figure 1B). Similarly, the MRI+ group had significantly higher IL-6 concentrations compared to the control group (*p* < 0.001). TNFα was significantly higher in the CT+ group compared to the MRI+ (*p* = 0.013) and control (*p* < 0.001) groups (Figure 1C); the MRI+ and control groups did not significantly differ (*p* = 0.444). Concentrations of VEGF were significantly higher in the CT+ than both the MRI+ (*p* < 0.001) and control (*p* < 0.001) groups; as well significantly higher in MRI+ compared to controls (*p* = 0.01) (Figure 1A). Concentrations of IL-10 did not differ between groups (Figure 1D). The IL-6/IL-10 ratio was significantly higher in the CT+ group compared to both the control (*p* < 0.001) and the MRI+ groups (*p* = 0.092); the MRI+ group compared with controls (p = 0.072) did not significantly differ. Age, sex, hyperlipidemia and hypertension were not significant predictors in any of the ANCOVA models.

**Figure 2:**
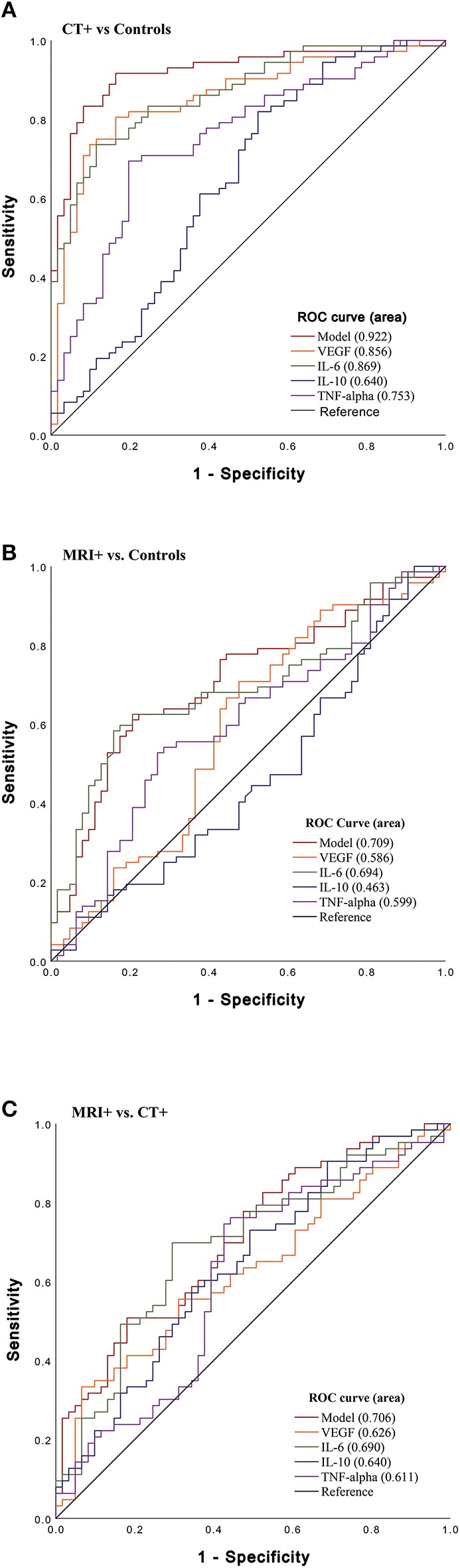
Sensitivity of Acute Cytokines to Predict Imaging Group. Receiver operating characteristic (ROC) curves for VEGF, IL-6, IL-10, and TNFα and combined model which includes all biomarkers (VEGF, IL-6, IL-10, TNFα). **(A)** ROC stratifying CT+ patients vs. controls (CT– and MRI–) [VEGF (AUC 0.86, 95% CI 0.80–0.92); IL-6 (AUC 0.87, 95% CI 0.81–0.93); TNFα (AUC 0.75, 95% CI 0.67–0.84); model (AUC 0.92, 95% CI 0.87–0.97)], **(B)** ROC stratifying MRI+ patients vs. controls [VEGF (AUC 0.59, 95% CI 0.49–0.69); IL-6 (AUC 0.70, 95% CI 0.60–0.78); and TNFα (AUC 0.60, 95% CI 0.50–0.73)] **(C)** ROC stratifying MRI+ patients vs. CT+ [VEGF (AUC 0.63, 95% CI 0.53–0.72), IL-6 (AUC 0.69, 95% CI 0.60–0.78), TNFα (AUC 0.61, 95% CI 0.51–0.71); model (AUC 0.71, 95% CI 0.62–0.80)].

To determine the ability of the cytokines to differentiate imaging groupings, an area under the curve (AUC) analysis was performed (Figure 2). In stratifying CT+ and controls, VEGF, IL-6, and TNFα were significant predictors and had fair to good AUC values (Figure 2A); the combined biomarker model showed good discriminatory power (AUC 0.92, 95% CI 0.87–0.97). In stratifying MRI+ and control groups, only IL-6 was a fair predictor (AUC 0.70, 95% CI 0.60–0.78; Figure 2B). Lastly, stratifying CT+ groups, VEGF, IL-6, and TNFα discriminated between these groups, and the combined model had a fair ability to distinguish groups (AUC 0.71, 95% CI 0.62–0.80; Figure 2C).

## Discussion

In this study, we report that plasma levels of IL-6, TNF-α, and VEGF are elevated acutely in patients with neuroimaging findings (CT or MRI) following a mTBI, and that these elevations remain significant after controlling for demographic and clinical factors. These findings are important, as they indicate that inflammatory cytokines are elevated within 24 h after mTBI, and that these elevations likely reflect neuronal damage resulting from the TBI that can be measured using either MRI or CT. These findings suggest that inflammatory activity relates to even the most mild neuronal injuries as determined by MRI. Further, total NSI scores were correlated with neuroimaging findings, but not inflammatory cytokines, in this cohort. Other mTBI studies have reported associations between postconcussive symptoms and inflammatory markers (31, 33). However, these studies report longer-term follow up on symptoms, while the present findings report one timepoint within 24 h after injury. In addition to limitations presented with self-reported symptoms, these findings should be examined in additional mTBI cohorts over time.

Proinflammatory cytokines, such as IL-6 and TNFα, are elevated after acute TBI and are also elevated in CVD patients (24, 27), yet the impact of these elevations, if experienced concomitantly, remain undetermined. To our knowledge, this is the first study to consider the possible impact of CVD risk factors on peripheral measures of inflammation following a mTBI. Interestingly, the present results suggest that CVD risk factors do not impact peripheral measures of IL-6, TNFα, or VEGF in the 24 h following mTBI, suggesting these biomarkers may have clinical utility, including in patients with CVD risk factors, though larger cohorts and temporal measures are needed to replicate this finding.

VEGF plays a central role in neurovascular health through processes that regulate angiogenesis, neurogenesis, neuroprotection, and astroglial proliferation following a TBI (39, 40, 44). Importantly, VEGF modulates inflammatory processes including cytokines and chemokines, and in turn, activates proinflammatory processes necessary for neuronal repair following a TBI (45, 46). Thus, our finding of increased VEGF levels in both MRI+ and CT+ patients compared to controls, suggests that VEGF in the peripheral blood reflects central activities that are related to recovery from a mTBI. This novel finding after acute mTBI indicates a possible mechanism for the modulation of inflammation, together with neurovascular effects, that should be further explored with symptomology and outcome measures.

A peripheral biomarker approach may be valuable to clinical care to improve detection of patients with mild injuries and to identify avenues for possible therapeutic interventions. Up to 25–40% of CT negative mTBI patients have positive MRI with increased likelihood of developing neurological symptoms (47, 48). In the present study, the combination of cytokines yields increased discriminatory power between subgroups, suggesting the need for additional, larger studies to identify the clinical utility of a multimarker approach, especially in the ability to stratify CT+ vs. MRI+(CT–). Additionally, further studies are needed to fully elucidate the role of the inflammatory response and links with neuroimaging in mTBI since inflammatory pathways are a promising avenue for possible future therapeutic interventions (40).

There are some limitations in this study. Because the sampling method was a convenience sample of participants who entered emergency departments in the DC metro area and volunteered to participate, results may not be generalizable to the entire mTBI population. The control patients (CT– MRI–) experienced external force trauma and there may be outcome deficits as well as subclinical biomarkers alterations even in this population with negative neuroimaging as observed in a previous report (49). Future work that includes comparison to a polytrauma control group, without head injuries, would be beneficial to elucidate the influence of peripheral cytokines exclusive to head injury in contrast to other bodily injuries. Evidence suggests that temporal peaks in peripheral inflammatory cytokines, including IL-6 and TNFα, occur within 24 h following injury, with some variation among studies (14, 50, 51). As such, an important direction for future work is the inclusion of additional time points in larger samples to map more precise temporal cascades for peripheral inflammatory response. Although outside of the scope of this study, an interesting future direction in larger studies will be to compare inflammatory markers with specific lesion types.

## Conclusion

In summary, our results suggest that IL-6, TNFα, and VEGF are promising biomarkers of brain injury in patients with acute mTBI. Therefore, a multi-biomarker approach, which includes inflammatory markers, may provide important insights into the mechanisms that relate to recovery from a mTBI. These findings are limited by a cross-sectional design in a relatively small population with only one timepoint. Our findings suggest that larger prospective studies are needed to evaluate implications of these findings on chronic symptomology and outcomes. Thus, there is a continued need for research to elucidate the biomarkers and subsequent underlying biological mechanisms which are involved in recovery, or lack thereof, from mTBI.

## Data Availability Statement

The raw data supporting the conclusions of this article will be made available by the authors, without undue reservation, to any qualified researcher.

## Ethics Statement

The studies involving human participants were reviewed and approved by National Institutes of Health Intramural Institutional Review Board. The patients/participants provided their written informed consent to participate in this study.

## Author Contributions

KE, JG, LT, and LL contributed to the conception or design of the study. KE contributed to drafting of the work. KE and CP contributed to interpretation of the data. KE, CP, and JP contributed to analysis of the data. JG, CP, VG, JP, CM, CD, and TD contributed to revising critically for intellectual content. LL, LT, and CD contributed to acquisition of the data. All authors read and approved the submitted version and agree to be accountable for all aspects of the work in ensuring that questions related to the accuracy or integrity of any part of the work are appropriately investigated and resolved.

## Conflict of Interest

The authors declare that the research was conducted in the absence of any commercial or financial relationships that could be construed as a potential conflict of interest.
